# Does persistent hyperprolactinemia contribute to bone loss independently of estrogen deficiency in postmenopausal women?

**DOI:** 10.1007/s11102-026-01724-3

**Published:** 2026-07-03

**Authors:** Ali Tunc, Ilkin Muradov, Mehmet Umut Capar, Banu Betul Kocaman, Mayis Farajli, Sebnem Yogurtcu, Oguz Alp Baykal, Sabriye Sibel Taze, Emre Durcan, Serdar Sahin, Hande Mefkure Ozkaya, Pinar Kadioglu

**Affiliations:** 1 Division of Endocrinology, Metabolism and Diabetes, Department of Internal Medicine, Cerrahpasa Faculty of Medicine, Istanbul University- Cerrahpasa, Istanbul, Türkiye Turkey; 2 Department of Internal Medicine, Cerrahpasa Faculty of Medicine, Istanbul University-Cerrahpasa, Kocamustafapasa Street No:53, Istanbul, Türkiye 34098 Turkey; 3 Division of Endocrinology, Metabolism and Diabetes, Department of Internal Medicine Nursing Service, Istanbul University-Cerrahpasa, Istanbul, Türkiye Turkey

**Keywords:** Prolactinoma, Postmenopausal, Hyperprolactinemia, Bone mineral density, Dopamine agonist discontinuation, Osteoporosis

## Abstract

**Objective:**

To evaluate bone mineral density (BMD) in postmenopausal women with prolactinoma diagnosed during the premenopausal period and to compare skeletal outcomes according to prolactin status during drug-free follow-up.

**Methods:**

This retrospective cross-sectional single-center study included 42 postmenopausal women with prolactinoma. Patients were classified as hyperprolactinemic or normoprolactinemic based on serum prolactin levels at the last follow-up visit. BMD was assessed by dual-energy X-ray absorptiometry at the lumbar spine and femoral neck. Clinical variables were compared between groups. Correlation analysis, receiver operating characteristic (ROC) analysis, and multivariable logistic regression were performed.

**Results:**

Lumbar spine BMD was significantly lower in hyperprolactinemic patients than in normoprolactinemic patients (0.861 ± 0.124 vs. 0.960 ± 0.129 g/cm², *p* = 0.022), whereas femoral neck BMD did not differ. Patients with hyperprolactinemia had longer cumulative exposure to hyperprolactinemia and required longer and higher-dose cabergoline treatment. Baseline prolactin levels were higher in patients with osteoporosis and showed potential discriminatory value for identifying osteoporosis (AUC = 0.861). Lumbar spine BMD and T-scores were inversely correlated with prolactin levels. In multivariable analysis, baseline prolactin level remained independently associated with low BMD.

**Conclusion:**

Persistent hyperprolactinemia is associated with lower lumbar spine BMD in postmenopausal women with prolactinoma. Baseline prolactin level and cumulative exposure to prolactin excess appear to be important contributors to skeletal deterioration, supporting the need for routine bone health assessment during long-term follow-up.

## Introduction

Prolactinoma is the most common functional pituitary adenoma, accounting for approximately 40% of all pituitary adenomas [1]. Its clinical manifestations arise primarily from hyperprolactinemia and the mass effect of the adenoma. The skeletal effects of prolactin extend well beyond its classical endocrine functions. While prolactin is primarily recognized for its role in mammary gland development and lactation, accumulating evidence has established its significant involvement in bone metabolism [2]. Studies in animal models have demonstrated that physiological prolactin concentrations are indispensable for normal bone growth and remodeling homeostasis. Complementary in vitro and experimental studies indicate that supraphysiological prolactin levels directly suppress osteoblastic activity and shift the RANKL/osteoprotegerin balance toward increased osteoclast activation, thereby enhancing bone resorption [3–5]. In the clinical setting of prolactinoma, this imbalance is further amplified; bone turnover is markedly increased, with resorption consistently exceeding formation [6]. Hyperprolactinemia also undermines skeletal integrity through an indirect pathway, namely the suppression of gonadal steroidogenesis secondary to hypogonadotropic hypogonadism [7]. The relative contribution of each mechanism to bone mineral density (BMD) loss, however, remains contested. Although some investigators attribute the predominant skeletal burden to gonadal insufficiency, a growing body of evidence supports a direct and independent detrimental role of hyperprolactinemia itself, independent of its effects on gonadal function [8, 9].

Postmenopausal women with prolactinoma represent a particularly vulnerable yet underrecognized clinical subgroup. In those in whom dopamine agonist therapy was discontinued during the postmenopausal period, the skeletal consequences of sustained prolactin excess on bone tissue remain incompletely characterized. Notably, postmenopausal women are already predisposed to osteoporosis and fragility fractures owing to estrogen deficiency; superimposed hyperprolactinemia may therefore constitute an additional and potentially underappreciated risk factor for accelerated bone loss and fracture susceptibility.

In routine clinical practice, elevated prolactin levels in postmenopausal women are frequently managed conservatively without pharmacological intervention. This practice is largely concordant with current clinical guidelines, which do not recommend dopamine agonist therapy in postmenopausal women with prolactinoma — particularly in the setting of microprolactinoma — given the absence of estrogen deficiency-related symptoms and the typically indolent natural history of the disease in this population [10, 11]. Consequently, these patients may remain untreated for extended periods, during which the potential skeletal consequences of chronic prolactin excess accumulate unmonitored, and the independent skeletal effects of prolactin excess beyond gonadal insufficiency risk being systematically overlooked. Against this background, the present study was designed to investigate whether hyperprolactinemia exerts an independent and direct adverse effect on bone mineral density in postmenopausal women — distinct from and beyond the confounding influence of gonadal insufficiency.

## Materials and methods

### Study design and participants

This retrospective cross-sectional, single-center study was conducted at the Endocrinology, Metabolism and Diabetes Outpatient Clinic of Istanbul University–Cerrahpaşa, Cerrahpaşa Medical Faculty, between October 2024 and November 2025.

The study included patients who had been diagnosed with prolactinoma during the premenopausal period, subsequently entered the postmenopausal period, and were being followed for prolactinoma without receiving dopamine agonist therapy at the last follow-up visit in the postmenopausal period.

Patients were eligible for inclusion if they: (a) had been diagnosed with prolactinoma, (b) were postmenopausal (≥ 12 months of amenorrhea), (c) were under regular follow-up for at least 12 months, and (d) had not received dopamine agonist therapy at the last follow-up visit.

The exclusion criteria were: (a) causes of hyperprolactinemia other than prolactinoma, (b) growth hormone (GH) co-secretion, (c) presence of secondary causes of osteoporosis, (d) irregular follow-up, and (e) incomplete medical records.

GH co-secretion was excluded based on age-adjusted insulin-like growth factor-1 (IGF-1) levels, which were routinely measured in all patients alongside serum prolactin as part of our institutional protocol. Oral glucose tolerance test-based GH suppression testing was performed when clinically indicated. Patients with pathological findings consistent with GH/prolactin co-secreting pituitary adenomas were excluded.

## Measures

### Clinical and biochemical assessments

Sociodemographic and clinical data were systematically retrieved from medical records and supplemented by structured patient interviews. The following variables were recorded: age at diagnosis, age at menopause, time since menopause, total disease duration, cumulative duration of hyperprolactinemia, body mass index (BMI), smoking and alcohol use, history of surgical and medical treatment, duration since discontinuation of dopamine agonist therapy, concomitant biochemical parameters, and comorbid conditions.

To comprehensively evaluate biochemical status and bone metabolism, serum prolactin, follicle-stimulating hormone (FSH), luteinizing hormone (LH), estradiol, calcium, phosphorus, magnesium, alkaline phosphatase, parathyroid hormone, and 25-hydroxyvitamin D levels were measured as part of routine biochemical assessment. Serum prolactin levels were measured using the Elecsys Prolactin II assay on the Roche Cobas e601 analyzer (Roche Diagnostics GmbH, Mannheim, Germany) by electrochemiluminescence immunoassay (ECLIA), with intra-assay and inter-assay coefficients of variation below 4%. Serum prolactin levels obtained at the last follow-up visit were recorded, and patients were classified as hyperprolactinemic (> 25 ng/mL) or normoprolactinemic according to the laboratory-specific reference range.

Bone turnover markers were assessed in patients for whom serum samples were available at the visit during which DXA was performed (*n* = 32). Venous blood samples were collected under standardized fasting conditions, centrifuged promptly, and stored at appropriate temperatures prior to biochemical analysis. Serum β-CrossLaps (β-CTx), a marker of osteoclast-mediated bone resorption, and osteocalcin, a marker of osteoblastic bone formation, were measured using the Elecsys β-CrossLaps/serum and Elecsys N-MID Osteocalcin assays on the Roche Cobas e601 analyzer (Roche Diagnostics GmbH, Mannheim, Germany). For β-CTx, the intra-assay and inter-assay coefficients of variation were 1.5% and 1.7%, respectively; for osteocalcin, these were 1.1% and 2.0%, respectively. The reference range for β-CTx in postmenopausal women was 177–1015 pg/mL, and for osteocalcin 15–46 ng/mL.

## Bone mineral density assessment

Bone mineral density was measured at the last follow-up visit using dual-energy X-ray absorptiometry (DXA) (Hologic Inc., Marlborough, MA, USA). Patients were classified according to World Health Organization criteria as having normal bone mineral density (T-score ≥ − 1.0), osteopenia (T-score between − 1.0 and − 2.5), or osteoporosis (T-score ≤ − 2.5). 

### Ethical approval

The study protocol was approved by the Ethics Committee of Istanbul University-Cerrahpaşa, Cerrahpaşa Faculty of Medicine (E-83460662-050.04-1513310). The study was conducted in accordance with the Declaration of Helsinki. Written informed consent was obtained from all participants. 

### Statistical analysis

Statistical analyses were performed using SPSS version 26.0. The distribution of continuous variables was assessed using the Shapiro–Wilk normality test. Continuous variables with a normal distribution were expressed as mean ± standard deviation (SD), whereas non-normally distributed variables were presented as median [interquartile range (IQR)]. Comparisons between groups were performed using the Student’s t-test for normally distributed variables and the Mann–Whitney U test for non-normally distributed variables. Correlations between continuous variables were evaluated using the Pearson correlation test or the Spearman rank correlation test, as appropriate. To assess the diagnostic performance of serum prolactin levels at the time of diagnosis for identifying osteoporosis, ROC curve analysis was performed, and the area under the curve (AUC) with 95% confidence intervals was calculated. Multivariable logistic regression analysis was conducted to determine independent predictors of low bone mineral density (BMD). The dependent variable was low BMD status, defined as the presence of osteopenia or osteoporosis according to WHO criteria (normal BMD = 0; osteopenia/osteoporosis = 1). Baseline prolactin level at diagnosis, time since menopause, maximum weekly cabergoline dose, age, duration of hyperprolactinemia, and FSH, LH, and estradiol levels at the last follow-up visit were included as covariates in the model. Results were reported as odds ratios (ORs) with 95% confidence intervals (CIs). Model calibration was assessed using the Hosmer–Lemeshow goodness-of-fit test. All statistical tests were two-tailed, and a p value <0.05 was considered statistically significant. 

## Results

### Baseline clinical and demographic characteristics

The study population comprised 42 women with prolactinoma diagnosed during the premenopausal period who were postmenopausal at the time of evaluation. The median current age was 53.5 years [51–59], while the mean ages at diagnosis and menopause were 40.1 ± 7.2 and 48.6 ± 5.5 years, respectively. The median time since menopause was 4 years [2–9]. At initial presentation, menstrual irregularities (56.4%) and galactorrhea (43.6%) were the most common symptoms, followed by headache (35.9%), whereas visual disturbance was rare (2.6%). Infertility was uncommon (5.1%).

The cohort was characterized by a predominantly overweight to obese phenotype, with a mean body mass index of 30.9 ± 6.1 kg/m². Metabolic comorbidities included obesity (60.5%), dyslipidemia (75.6%), diabetes mellitus (12.2%), and hypertension (17.9%). Smoking was reported in 21.4% of patients, whereas no alcohol use was reported.

Biochemically, prolactin levels declined substantially from diagnosis to the time of evaluation, with median values of 105 ng/mL [71–195] and 16 ng/mL [10–31], respectively. Baseline FSH, LH, and estradiol levels were 5.26 [4.80–6.49] mIU/mL, 3.49 [2.22–4.44] mIU/mL, and 37.0 [21.0–43.0] pg/mL, respectively. At the last follow-up visit, mean FSH and LH levels were 66.8 ± 38.9 mIU/mL and 33.0 ± 18.0 mIU/mL, respectively, while median estradiol level was 5.6 pg/mL [5–14.75]. Serum calcium, phosphorus, magnesium, and alkaline phosphatase levels were within normal ranges. In patients with available measurements, mean β-CTX and osteocalcin levels were 455.9 ± 184.4 pg/mL and 23.1 ± 7.1 ng/mL, respectively. Median vitamin D levels were 24 ng/mL [21–33], within the insufficient range, while parathyroid hormone levels remained within normal limits.

Radiological evaluation demonstrated a median tumor size of 7 mm [5–9] at diagnosis. Cavernous sinus invasion and cystic tumor components were observed in 15% and 10% of patients, respectively.

Bone mineral density assessment revealed a heterogeneous skeletal profile, with osteopenia and osteoporosis present in 40.5% and 19% of patients, respectively. Mean lumbar spine BMD was 0.923 ± 0.134 g/cm² (T-score −1.2 ± 1.2), whereas mean femoral neck BMD was 0.799 ± 0.123 g/cm² (T-score −0.8 ± 1.0).

Patients had received long-term dopamine agonist therapy prior to treatment withdrawal, with a mean duration of 84 ± 50 months. The median maximum weekly cabergoline dose was 1 mg [0.5–1], and the median cumulative dose was 108 mg [46–325]. The median time since treatment discontinuation was 48 months [26–117]. The mean duration of disease was 191 ± 83 months, while the median duration of hyperprolactinemia was 41 months [11–96]. 

### Comparison of hyperprolactinemic and normoprolactinemic patients

Of the 42 patients, 26 (61.9%) were normoprolactinemic, whereas 16 (38.1%) were hyperprolactinemic.

The most striking difference between groups was in prolactin levels. Patients with hyperprolactinemia had significantly higher prolactin concentrations both at diagnosis (177 [104–228] vs. 86 [52–142] ng/mL, p = 0.006) and at the last follow-up visit (37 [28–63] vs. 11 [8–15] ng/mL, p<0.001) compared with normoprolactinemic patients.

Baseline gonadotropin and estradiol levels were comparable between groups. Median FSH levels were 5.0 [4.8–5.9] mIU/mL in hyperprolactinemic patients and 6.2 [5.0–7.4] mIU/mL in normoprolactinemic patients (p = 0.445). Median LH levels were 3.8 [2.6–4.4] and 3.0 [2.1–5.4] mIU/mL (p = 0.573), while median estradiol levels were 39.0 [30.0–42.5] and 30.5 [15.8–41.5] pg/mL (p = 0.714), respectively. Gonadotropin and estradiol levels at the last follow-up visit also remained comparable between groups. Mean FSH levels were 57.9 ± 26.8 mIU/mL in hyperprolactinemic patients and 72.2 ± 44.4 mIU/mL in normoprolactinemic patients (p = 0.252). Mean LH levels were 30.8 ± 14.3 and 34.3 ± 20.1 mIU/mL (p = 0.549), while median estradiol levels were 9.1 [5.0–13.9] and 5.5 [5.0–15.9] pg/mL (p = 0.864), respectively. Tumor size at diagnosis and current tumor size did not differ significantly between groups (7 [6–8] vs. 7 [5–12] mm, p = 0.855; and 6 [4.5–8] vs. 5 [3–6] mm, p = 0.283, respectively), despite the observed differences in prolactin levels.

Treatment history also differed substantially between groups. Hyperprolactinemic patients had received a longer duration of cabergoline therapy (p = 0.010), a higher maximum weekly dose (p = 0.035), and a higher cumulative cabergoline dose (p = 0.044), while the time since cabergoline withdrawal was shorter in this group compared with those who achieved normoprolactinemia (p = 0.047). Taken together, these data suggest that patients who remained hyperprolactinemic despite treatment represent a more refractory subgroup, characterized by greater disease burden and prolonged exposure to elevated prolactin levels. Five patients (11.9%) had previously undergone transsphenoidal surgery. Of these, four were normoprolactinemic and one was hyperprolactinemic at the last follow-up visit. Given the limited number of surgically treated patients, no formal subgroup analysis according to surgical history was performed.

Crucially, this sustained hyperprolactinemia appears to have left a measurable imprint on the skeleton. Lumbar spine BMD was significantly lower in the hyperprolactinemic group (0.861 ± 0.124 vs. 0.960 ± 0.129 g/cm², p = 0.022). Although femoral neck BMD and T-scores did not differ significantly between groups, the directional trend was consistent. Bone turnover markers — β-CTX (464.7 ± 229.9 vs. 449.9 ± 152.4 pg/mL, p = 0.828) and osteocalcin (22.3 ± 7.9 vs. 23.6 ± 6.7 ng/mL, p = 0.636) — were comparable between groups (Table 1).

**Table 1:** Comparison of Hyperprolactinemic and Normoprolactinemic Patients

Parameters	Hyperprolactinemic group (*n* = 16)	Normoprolactinemic group (*n* = 26)	*P* value
Demographic Characteristics			
Current age (years, median [IQR])	53.5 [50.3–59.8]	53.5 [51.8–59]	0.866
Age at diagnosis (years, mean ± SD)	37.8 ± 9.4	41.5 ± 5.2	0.116
Menopause age (years, mean ± SD)	50.3 ± 4.5	47.6 ± 5.9	0.138
Time since menopause (years, median [IQR])	3 [2–9]	4 [2–10]	0.452
**Tumor Characteristics**			
Tumor size at diagnosis (mm, median [IQR])	7 [6–8]	7 [5–12]	0.855
Current tumor size (mm, median [IQR])	6 [4.5–8]	5 [3–6]	0.283
**Prolactin Levels**			
Baseline prolactin level (ng/mL, median [IQR])	177 [104–228]	86 [52–142]	**0.006**
Current prolactin level (ng/mL, median [IQR])	37 [28–63]	11 [8–15]	**< 0.001**
**Cabergoline Treatment Characteristics**			
Cabergoline treatment duration (months, mean ± SD)	110 ± 51	68 ± 44	**0.010**
Maximum weekly cabergoline dose (mg/week, median [IQR])	1 [0.5–3]	0.5 [0.5–1]	**0.035**
Cumulative cabergoline dose (mg, median [IQR])	288 [48–399]	92 [43–181]	**0.044**
Time since cabergoline withdrawal (months, median [IQR])	33 [16–72]	59 [43–120]	**0.047**
**Disease Duration Parameters**			
Duration of illness (months, mean ± SD)	206 ± 98	182 ± 74	0.377
Duration of hyperprolactinemia (months, median [IQR])	108 [47–163]	20 [5–50]	**< 0.001**
**Anthropometric Parameters**			
BMI (kg/m², mean ± SD)	30.4 ± 5.7	31.2 ± 6.5	0.685
**Bone Mineral Density Parameters**			
Lumbar spine BMD (g/cm², mean ± SD)	0.861 ± 0.124	0.960 ± 0.129	**0.022**
Lumbar spine T-score (mean ± SD)	−1.6 ± 1.1	−0.9 ± 1.1	0.051
Femoral neck BMD (g/cm², mean ± SD)	0.761 ± 0.132	0.822 ± 0.114	0.122
Femoral neck T-score (mean ± SD)	−1.0 ± 1.0	−0.6 ± 1.0	0.193
**Bone Turnover Markers**			
β-CrossLaps (β-CTX) (pg/mL, mean ± SD)	464.7 ± 229.9	449.9 ± 152.4	0.828
Osteocalcin (ng/mL, mean ± SD)	22.3 ± 7.9	23.6 ± 6.7	0.636

*BMI* Body mass index, *BMD* Bone mineral density, *IQR* Interquartile range, *SD* Standard deviation

### Clinical and biochemical characteristics according to DXA categories

When categorical clinical variables were evaluated according to bone mineral density categories, no significant associations were observed (Table 2). In contrast, several continuous parameters differed significantly across DXA groups (Table 3).Age differed significantly across groups (p = 0.009). Post-hoc analysis showed that patients in the osteoporotic group were significantly older than both the normal (adjusted p = 0.035) and osteopenic groups (adjusted p = 0.008), while no significant difference was observed between the normal and osteopenic groups (adjusted p = 1.000). Similarly, time since menopause differed significantly among DXA groups (p = 0.013), with a longer time since menopause in the osteoporosis group compared with the normal group (adjusted p = 0.011).

**Table 2:** Categorical Clinical and Tumor-Related Characteristics According to BMD Categories

Variable	Normal, *n* (%)	Osteopenia, *n* (%)	Osteoporosis, *n* (%)	*P* value
Hyperprolactinemia	5 (31.3)	5 (31.3)	6 (37.5)	0.058
Smoker	4 (44.4)	4 (44.4)	1 (11.2)	0.940
Headache at diagnosis	4 (28.6)	7 (50.0)	3 (21.4)	0.273
Visual impairment at diagnosis	0 (0)	1 (100.0)	0 (0)	0.515
Menstrual irregularity at diagnosis	10 (45.5)	8 (36.4)	4 (18.2)	0.411
Galactorrhea at diagnosis	9 (52.9)	6 (35.3)	2 (11.8)	0.575
Infertility at diagnosis	2 (100.0)	0 (0)	0 (0)	0.256
Cavernous sinus invasion (initial MRI)	2 (33.3)	4 (66.7)	0 (0)	0.338
Cystic component (initial MRI)	1 (25.0)	3 (75.0)	0 (0)	0.344
Obesity	11 (47.8)	9 (39.1)	3 (13.0)	0.504

Data are presented as n (%). Only one category per variable is shown; the complementary category was considered the reference group. *BMD* Bone mineral density, MRI magnetic resonance imaging

**Table 3:** Clinical, Biochemical, and Treatment Characteristics According to BMD Categories

Variable	Normal (*n* = 17)	Osteopenia (*n* = 17)	Osteoporosis (*n* = 8)	*P* value
Demographic Characteristics				
Current age (years, median, IQR)	54 [50.5–57.5]	52 [51–54.5]	59.5 [58.3–64.3]	**0.009**
Age at diagnosis (years, mean ± SD)	41.1 ± 5.5	39.6 ± 7.7	39.0 ± 10.1	0.765
Menopause age (years, mean ± SD)	49.7 ± 4.1	46.7 ± 6.3	50.4 ± 5.9	0.196
Time since menopause (years, median, IQR)	2.5 [1–6.8]	3 [2–10]	10 [9–10]	**0.013**
**Tumor Characteristics**				
Tumor size at diagnosis (mm, median, IQR)	7 [5.3–8]	7 [5–11.5]	8 [7–14]	0.435
Current tumor size (mm, median, IQR)	4.8 [2.8–5.5]	5 [3.2–11.5]	7.0 [6–7]	0.464
**Prolactin Levels**				
Baseline prolactin level (ng/mL, median, IQR)	82 [69–117]	105 [49–175]	219 [164–470]	**0.009**
Current prolactin level (ng/mL, median, IQR)	15 [10–33]	15 [8–25]	39 [18–63]	0.069
**Cabergoline Treatment Characteristics**				
Cabergoline treatment duration (months, mean ± SD)	70 ± 47	89 ± 54	105 ± 45	0.266
Maximum weekly cabergoline dose (mg/week, median, IQR)	1.0 [0.5–1.3]	0.6 [0.5–1]	1.5 [1–3.5]	**0.047**
Cumulative cabergoline dose (mg, median, IQR)	94 [52–331]	106 [34–295]	300 [102–764]	0.247
Time since cabergoline withdrawal (months, median, IQR)	54 [20–92]	43 [28–105]	90 [33–144]	0.399
**Disease Duration Parameters**				
Duration of illness (months, mean ± SD)	174 ± 76	189 ± 83	237 ± 92	0.249
Duration of hyperprolactinemia (months, median, IQR)	32 [10–89]	37 [10–82]	129 [27–163]	0.152
**Anthropometric Parameters**				
BMI (kg/m², mean ± SD)	30.7 ± 4.6	31.7 ± 7.5	29.4 ± 6.7	0.707
**Biochemical Parameters**				
PTH (pg/mL, median, IQR)	38 [28–46]	44 [30–53]	34 [28–61]	0.678
Vitamin D (ng/mL, median, IQR)	24 [21–32]	24 [20–31]	28 [21–38]	0.679

*BMI* Body mass index, *DXA* Dual-energy X-ray absorptiometry, *BMD* Bone mineral density, *PTH* Parathyroid hormone, *IQR* Interquartile range, *SD* Standard deviation

Prolactin levels at diagnosis differed significantly across DXA categories (p = 0.009). Post-hoc analysis confirmed that patients who developed osteoporosis had significantly higher prolactin levels at diagnosis compared with both the normal (adjusted p = 0.007) and osteopenic groups (adjusted p = 0.040).

Baseline gonadotropin and estradiol levels were also evaluated across DXA categories. FSH levels did not differ significantly across groups (normal: 4.5 [4.2–5.5], osteopenia: 6.2 [5.0–9.8], osteoporosis: 5.2 [5.1–5.2] mIU/mL; p = 0.349). Estradiol levels were similarly comparable (normal: 39.0 [31.5–41.0], osteopenia: 41.5 [31.0–57.3], osteoporosis: 15.5 [12.8–18.3] pg/mL; p = 0.211). LH levels differed significantly across DXA categories (normal: 2.3 [2.2–2.6], osteopenia: 4.6 [3.9–15.6], osteoporosis: 1.7 [1.6–1.8] mIU/mL; p = 0.006); post-hoc analysis revealed that this difference was driven by significantly higher LH levels in the osteopenic group compared to the normal BMD group (p = 0.008), while no significant difference was observed between the osteoporotic and normal BMD groups (p = 1.000) or between the osteoporotic and osteopenic groups (p = 0.167). Gonadotropin and estradiol levels at the last follow-up visit were also comparable across DXA categories. FSH levels were 72.7 ± 36.7, 68.1 ± 44.7, and 51.3 ± 29.4 mIU/mL in the normal, osteopenic, and osteoporotic groups, respectively (p = 0.443). LH levels were 37.7 ± 18.7, 31.9 ± 18.2, and 25.3 ± 15.0 mIU/mL (p = 0.270), while median estradiol levels were 12.4 [5.0–17.6], 5.6 [5.0–13.7], and 5.0 [5.0–10.0] pg/mL (p = 0.551).

The maximum weekly cabergoline dose also differed significantly across groups (p = 0.047) and was higher in osteoporotic patients compared with those with osteopenia (adjusted p = 0.040). Two patients in the osteoporotic group were receiving bisphosphonate therapy at the time of DXA assessment, both of whom were in the hyperprolactinemic group. In one patient, lumbar spine BMD could not be evaluated due to a vertebral fracture, and only femoral neck measurements were available. All other clinical, anthropometric, metabolic, and hormonal parameters were comparable across DXA categories.

**Fig. 1:**
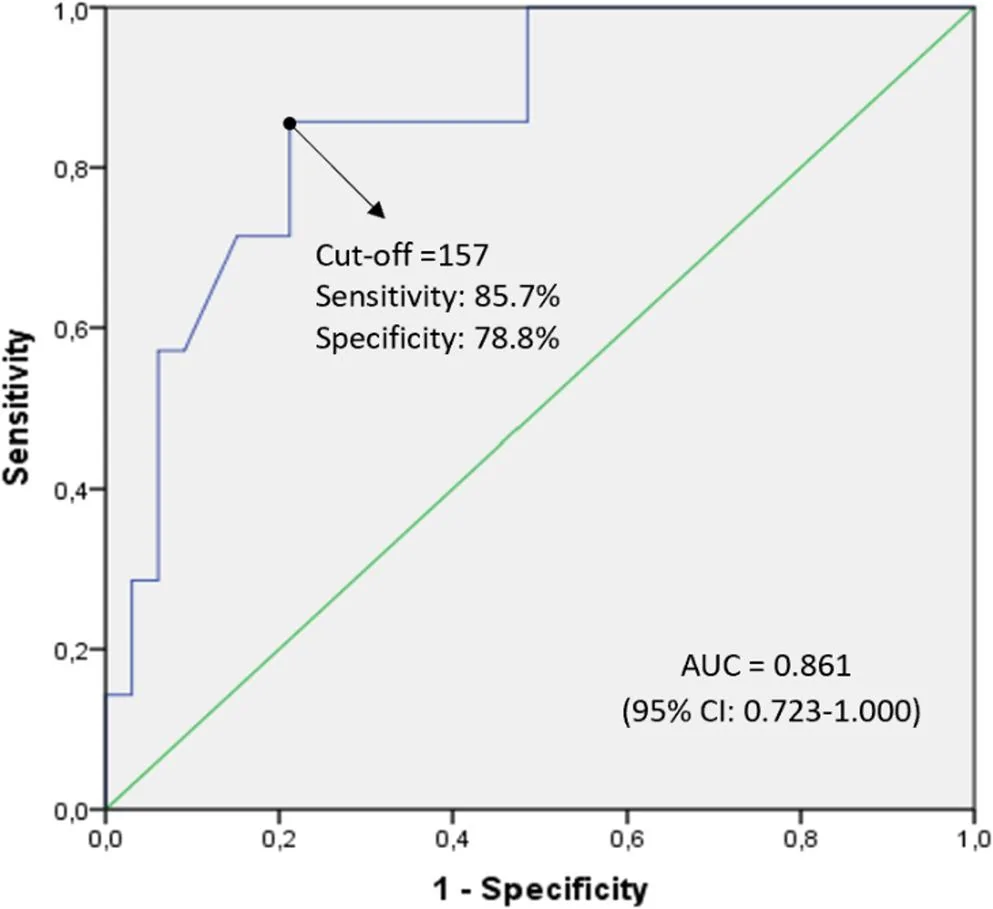
Receiver operating characteristic (ROC) curve showing the discriminatory ability of baseline prolactin levels for identifying osteoporosis. The area under the curve (AUC) was 0.861 (95% confidence interval: 0.723–1.000). A cut-off value of 157 yielded a sensitivity of 85.7% and a specificity of 78.8%

The discriminatory ability of prolactin levels at diagnosis for identifying osteoporosis was assessed using ROC analysis (Fig. 1). The area under the curve (AUC) was 0.861 (standard error: 0.070; 95% confidence interval: 0.723–1.000; p = 0.003). Analysis of the ROC curve coordinates showed that a cut-off value of 157 ng/mL yielded a sensitivity of 85.7% and a specificity of 78.8%. 

### Correlation analysis

Correlation analyses revealed a consistent and biologically coherent pattern linking prolactin excess to skeletal deterioration. Lumbar spine T-score was inversely correlated with prolactin levels at diagnosis (r = −0.445, p = 0.005), and lumbar spine BMD showed negative correlations with both prolactin levels at diagnosis (r = −0.409, p = 0.011) and current prolactin levels (r = −0.325, p = 0.041) — reinforcing the hypothesis that the duration and magnitude of prolactin exposure cumulatively impair axial bone mass.

Femoral neck parameters told a complementary story. Femoral neck T-score was negatively correlated with current age (r = −0.352, p = 0.028), duration of cabergoline treatment (r = −0.338, p = 0.035), and cumulative cabergoline dose (r = −0.354, p = 0.025). Femoral neck BMD was similarly negatively correlated with cumulative cabergoline dose (r = −0.346, p = 0.029) and current prolactin levels (r = −0.345, p = 0.025). Notably, femoral neck BMD was positively correlated with estradiol levels measured at the last follow-up visit (r = 0.418, p = 0.007). 

### Multivariable analysis

In the multivariable logistic regression analysis, baseline prolactin level at diagnosis remained independently associated with low BMD (OR = 1.016; 95% CI: 1.001–1.030; p = 0.031). Age (p = 0.145), time since menopause (p = 0.229), duration of hyperprolactinemia (p = 0.081), maximum weekly cabergoline dose (p = 0.125), and FSH (p = 0.184), LH (p = 0.324), and estradiol levels (p = 0.280) at the last follow-up visit were not independently associated with low BMD (Table 4). 

**Table 4:** Multivariable logistic regression analysis for predictors of low BMD

Parameters	OR	95% CI	*P* value
Current age (years)	0.786	0.568–1.090	0.145
Time since menopause (years)	1.176	0.903–1.530	0.229
Baseline prolactin level (ng/mL)	1.016	1.001–1.030	**0.031**
Duration of hyperprolactinemia (months)	1.024	0.997–1.050	0.081
Maximum weekly cabergoline dose (mg/week)	0.278	0.054–1.430	0.125
FSH at last follow-up (mIU/mL)	1.046	0.979–1.120	0.184
LH at last follow-up (mIU/mL)	0.920	0.780–1.090	0.324
Estradiol at last follow-up (pg/mL)	0.950	0.867–1.040	0.280

*OR* Odds ratio, *CI* Confidence interval, *BMD* Bone mineral density

## Discussion

In this study, we evaluated bone mineral density outcomes in 42 postmenopausal women with prolactinoma who were diagnosed during the premenopausal period and underwent dopamine agonist treatment withdrawal. Our findings show that patients with persistent hyperprolactinemia after menopause had significantly lower lumbar spine BMD compared with those who achieved normoprolactinemia, despite similar age, menopause age, and time since menopause between groups. Higher prolactin levels at diagnosis were associated with vertebral bone loss and showed potential discriminatory value for identifying osteoporosis on ROC analysis. In addition, baseline prolactin level at diagnosis remained independently associated with low BMD in multivariable logistic regression analysis.

These findings challenge the prevailing clinical view that prolactinoma becomes less relevant after menopause. While management has traditionally focused on reproductive consequences and often becomes more conservative after menopause, our results indicate that persistent hyperprolactinemia is associated with lower lumbar spine BMD even in the postmenopausal period. This suggests that the skeletal impact of prolactin excess may extend beyond reproductive function and remain clinically relevant after menopause.

Prolactin exerts multiple extragonadal and metabolic effects that extend beyond the hypothalamic–pituitary–gonadal axis [12–15]. Experimental and clinical evidence suggests that hyperprolactinemia may adversely affect bone remodeling independently of gonadal status, contributing to increased bone resorption and vertebral fracture risk [4,16]. It should be emphasized, however, that most existing evidence originates from premenopausal cohorts, and data specifically addressing the postmenopausal setting remain limited. Our findings extend this evidence by suggesting that prolactin-related skeletal effects remain clinically relevant after menopause. Although estrogen deficiency remains an important determinant of skeletal health, the observed association between prolactin exposure and low BMD persisted after accounting for gonadal hormonal status. Notably, however, hyperprolactinemia status was not significantly associated with DXA categories in the categorical analysis. This finding should be interpreted in the context of the limited sample size and the relatively small number of osteoporotic patients. In contrast, analyses based on continuous measures of prolactin exposure demonstrated consistent associations with lumbar spine BMD, osteoporosis risk, and low BMD in multivariable analysis. The apparent discrepancy may reflect the lower statistical power of categorical comparisons, particularly in a small cohort, as well as the limited ability of categorical analyses to capture the full spectrum of prolactin exposure.

Trabecular bone is particularly sensitive to hormonal disturbances and is therefore disproportionately vulnerable in endocrine disorders such as hyperprolactinemia, especially at vertebral skeletal sites where trabecular bone predominates. Consistent with this physiological framework, a comprehensive review on the skeletal effects of hyperprolactinemia reported that bone loss predominantly involves trabecular-rich sites, with more pronounced reductions in lumbar spine BMD compared with cortical sites [17]. Similarly, Schlechte et al. demonstrated that spinal bone mineral content was markedly reduced in hyperprolactinemic women, whereas cortical bone at the forearm was only minimally affected, indicating predominant involvement of trabecular bone in the axial skeleton [18]. Our findings align with this established pattern: lumbar spine BMD was significantly lower in patients with a hyperprolactinemic disease course, while femoral neck BMD did not differ significantly between groups. The negative correlations between prolactin levels and both lumbar spine BMD and T-scores further reinforce the association between prolactin exposure and vertebral bone loss in the postmenopausal setting.

Beyond the site-specific skeletal involvement observed at the lumbar spine, the chronicity and cumulative burden of hyperprolactinemia appear to be important contributors to long-term skeletal impairment. Mazziotti et al. observed higher prolactin levels in patients with vertebral fractures and identified disease duration as an independent factor associated with fracture risk, thereby emphasizing the role of chronic exposure to prolactin excess in vertebral skeletal deterioration [16]. Similarly, Andereggen et al. reported that persistent hyperprolactinemia was an independent risk factor for long-term bone impairment in a cohort of patients with prolactinoma and demonstrated that skeletal deterioration may persist despite biochemical control of prolactin levels and hypogonadism [19], suggesting that bone impairment reflects the cumulative burden of long-standing disease. In this context, although overall disease duration did not differ significantly between groups in our cohort, the duration of exposure to hyperprolactinemia was longer in patients with adverse skeletal outcomes, indicating that cumulative biochemical exposure may better reflect the true skeletal burden of the disease than chronological disease duration alone. Moreover, persistently elevated prolactin levels and the strong predictive value of baseline prolactin for osteoporosis further suggest that both the magnitude and duration of prolactin excess may have lasting effects on skeletal health. Taken together, these findings support the notion that prolonged exposure to prolactin excess may contribute to progressive vertebral bone deterioration in the postmenopausal period, alongside other established determinants of skeletal health.

Recent literature has expanded the understanding of skeletal involvement in prolactinoma beyond conventional DXA-derived bone mineral density measurements. Vertebral fractures may occur even in patients with normal or only mildly reduced BMD, suggesting that DXA alone may underestimate the true skeletal burden of prolactinoma-related bone disease [20]. Contemporary approaches increasingly emphasize the assessment of bone quality and trabecular microarchitecture using tools such as trabecular bone score (TBS), high-resolution peripheral quantitative computed tomography (HR-pQCT), and vertebral fracture assessment [21]. Recent studies have further demonstrated preferential involvement of trabecular-rich skeletal sites and increased vertebral fracture risk associated with prolonged hyperprolactinemia, even in the presence of preserved areal BMD [20]. In this context, our findings should be interpreted within this emerging framework of prolactinoma-related skeletal fragility, in which prolonged hyperprolactinemia may adversely affect trabecular bone and skeletal integrity beyond conventional DXA-derived BMD measurements alone.

Several limitations of the present study warrant consideration. First, the retrospective design and relatively small sample size limit causal inference and may affect the generalizability of findings to broader prolactinoma populations. Second, bone mineral density was assessed using DXA, which does not provide information on bone microarchitecture or vertebral fragility; accordingly, subtle alterations in bone quality and trabecular integrity may not have been fully captured. In addition, systematic morphometric vertebral fracture assessment was not performed in this cohort. Given that vertebral fractures may occur even in prolactinoma patients with normal or only mildly reduced BMD, DXA-based evaluation alone may underestimate the true skeletal burden associated with prolonged hyperprolactinemia. In addition, median vitamin D levels in the cohort were within the insufficient range, which may have contributed to the observed bone mineral density findings and should be considered when interpreting the skeletal outcomes. Two patients in the osteoporotic group were receiving bisphosphonate therapy at the time of DXA assessment. Bone turnover marker data were unavailable for these patients. As both were in the hyperprolactinemic group, bisphosphonate use may have partially attenuated BMD differences between groups, suggesting that the true skeletal deficit associated with persistent hyperprolactinemia may have been underestimated.

In postmenopausal women with prolactinoma undergoing drug-free follow-up, prolactin levels may remain normal in some patients, whereas others develop recurrent hyperprolactinemia after treatment withdrawal. Postmenopausal women with persistent hyperprolactinemia demonstrate significantly lower lumbar spine BMD, with baseline prolactin level remaining independently associated with low BMD in multivariable analysis and showing potential discriminatory value for identifying osteoporosis. Nevertheless, given the limited number of osteoporotic patients in our cohort, this ROC-derived threshold should be interpreted cautiously and requires validation in larger prospective studies. Prolonged biochemical exposure to prolactin excess appears to be an important contributor to skeletal deterioration in this population, alongside established postmenopausal skeletal risk factors. The higher doses and longer duration of cabergoline therapy, together with the shorter interval since treatment withdrawal observed in hyperprolactinemic patients, most likely reflect the refractory nature of the underlying disease and the associated clinical caution regarding dopamine agonist discontinuation. In addition, rebound hyperprolactinemia following treatment withdrawal should be considered as a contributing factor. As treatment withdrawal and drug-free follow-up represent the dominant clinical approach in postmenopausal prolactinoma patients, skeletal risk may be systematically underestimated — particularly in those with a history of persistent or more severe hyperprolactinemia. With this risk in mind, a more proactive management strategy should be considered in patients found to have osteoporosis or vertebral fractures, including continued dopamine agonist therapy in selected patients, optimization of vitamin D and calcium status, and bone-active therapies such as bisphosphonates when clinically indicated. Given the multifaceted nature of skeletal comorbidity in prolactinoma, multidisciplinary management within Pituitary Tumor Centers of Excellence (PTCOEs) may further optimize long-term skeletal outcomes [22]. Bone mineral density assessment should therefore be explicitly incorporated into long-term follow-up decisions in this population, regardless of whether dopamine agonist therapy is continued or withdrawn.

## Data Availability

The datasets generated and/or analyzed during the current study are available from the corresponding author on reasonable request.
